# Mild Anemia and Pregnancy Outcome in a Swiss Collective

**DOI:** 10.1155/2014/307535

**Published:** 2014-11-13

**Authors:** Gabriela Bencaiova, Christian Breymann

**Affiliations:** Division of Obstetrics, Department of Obstetrics and Gynecology, University Hospital of Zurich, Frauenklinikstrasse 10, 8091 Zurich, Switzerland

## Abstract

*Background*. Over half of all women in the world experience anemia during their pregnancy. Our aim was to investigate the relation between hemoglobin and iron status examined in second trimester and pregnancy outcome. *Methods*. In a prospective longitudinal study, 382 pregnant women were included. Blood samples were examined for hematological status and serum ferritin between 16 and 20 weeks and for hemoglobin before delivery. The adverse maternal and perinatal outcomes were determined. Regression analysis was performed to establish if anemia and low serum ferritin are risk factors for pregnancy complications. *Results*. There was no increase of complications in women with mild anemia and in women with depleted iron stores. The finding showed that mild iron deficiency anemia and depleted iron stores are not risk factors for adverse outcomes in iron supplemented women. *Conclusions*. Mild anemia and depleted iron stores detected early in pregnancy were not associated with adverse maternal and perinatal outcomes in iron supplemented women.

## 1. Introduction

Over half of all women in the world experience anemia during their pregnancy [1–4]. The association between the gestational age at which anemia is diagnosed and adverse pregnancy outcomes is an important issue [5, 6]. Some of the increase in anemia and iron deficiency anemia (IDA) with gestation is a consequence of the normal physiological changes of pregnancy [7]. To avoid the difficulties in anemia detection caused by plasma volume increase, the examination should be conducted until 20 weeks of gestation.

Findings from the studies on the relationship between anemia and adverse pregnancy outcome are contradictory. Several studies have shown that preterm delivery, small for gestational age, and low birth weight are increased for women with anemia during the 1st trimester and risk depends on the severity of the hemoglobin deficit [6, 8–11]. Women with hemoglobin between 8.0 and 9.9 g/dL had significantly higher risk for low birth weight, preterm birth, and small for gestational age than women with hemoglobin between 10.0 and 11.9 g/dL [12]. The observation by Scholl et al. showed that only iron deficiency anemia, not any other anemia, was related to preterm birth, which suggests that some iron specific mechanism may be at play [12].

Severe anemia is also associated with adverse maternal outcome and may contribute directly or indirectly to a significant proportion of maternal cardiac failure, hemorrhage, and infection. On the other hand, higher rates of placental problems (abnormal placentation and placental abruption) were found among the anemic women [13].

The aim of this study was to investigate the relationship between hemoglobin concentration and serum ferritin and adverse outcomes. The logistic regression analysis was performed to establish if anemia and low serum ferritin are risk factors for well-known adverse pregnancy outcome.

## 2. Methods

### 2.1. Study Population

A prospective longitudinal study was performed at the Department of Obstetrics, University Hospital of Zurich, to determine the relationship between hemoglobin concentration and serum ferritin and adverse outcome. The study was approved by the Human Research Ethics Committee at the Women's Hospital in Zurich. The women were asked for their consent to participate in our study and the informed consent was obtained before study enrolment.

The hematological status and serum ferritin were examined in 382 pregnant women between 16 and 20 pregnancy weeks and hemoglobin concentration before delivery. All women were presented with singleton pregnancies. Exclusion criteria included chronic renal disease and malignancies and having a blood transfusion at least 3 months before enrolment in the study. Women with hemoglobin (Hb) between 10.0 and 11.0 g/dL received oral iron supplementation. Women with hemoglobin <10.0 g/dL were treated directly with intravenous iron in the anemia clinic if they agreed with intravenous therapy.

### 2.2. Study Criteria

According to current guidelines based on recommendations of the CDC, anemia in pregnancy is defined by a hemoglobin value less than 11.0 g/dL in both the first and third trimesters and less than 10.5 g/dL in the second trimester [14]. On the basis of our experiences determining Hb (error of measurement of Hb ±0.5 g/dL) and the related high intraindividual variations, we chose Hb < 11.0 g/dL as the cut-off. Iron deficiency anemia was defined as Hb < 11.0 g/dL and a serum ferritin ≤ 15 *μ*g/L. Depleted iron stores was defined as a serum ferritin < 20 *μ*g/L. Anemia for other reasons was defined as Hb < 11.0 g/dL and ferritin > 15 *μ*g/L. The category anemia for other reasons included the following: thalassemia and hemoglobinopathies, vitamin B12 deficiency anemia, folic acid deficiency anemia, and chronic inflammatory diseases (particularly HIV positive women, active hepatitis B).

The women were divided according to hemoglobin concentration and ferritin levels into women with iron deficiency anemia (Hb < 11.0 g/dL and ferritin ≤ 15 *μ*g/L) (Group 1), women with depleted iron stores without anemia (Hb ≥ 11.0 g/dL and ferritin < 20 *μ*g/L) (Group 2), women with anemia for other reasons (Hb < 11.0 g/dL and ferritin > 15 *μ*g/L) (Group 3), and women with normal status (Group 4: control group).

### 2.3. Laboratory Assessment

Blood samples were collected by venipuncture. Hb, red blood cells (RBC), hematocrit (HCT), mean corpuscular volume (MCV), percentage of red cells, microcytic, macrocytic, hypochromic, and hyperchromic erythrocytes, hemoglobin content of reticulocytes (CHr), and red blood cell distribution width (RDW) were measured using an ADVIA hematology analyser system (Bayer Diagnostics, Leverkusen, Germany). Mean corpuscular hemoglobin (MCH) was automatically calculated from Hb and RBC. Ferritin was assessed by chemiluminescence immunoassay (ACS 190; Ciba/Corning Diagnostic Corp., Cleveland, OH).

### 2.4. Maternal and Perinatal Outcomes

Postpartum hemorrhage was defined as Hb decrease of more than 3.0 g/dL on the second day after delivery. Abnormal site of placental implantation (placenta praevia) and abnormal placental penetration (placenta accreta/increta/percreta) were described as abnormal placental invasion or abnormal placentation. Gestational age was determined on the basis of early ultrasound examination. Low birth weight (LBW) was defined as birth weight <2500 g. Preterm birth was defined as birth before 37 completed weeks of gestation. Preterm premature rupture of fetal membranes (PPROM) was defined as rupture of fetal membranes before 37 completed weeks of gestation. Intrauterine growth restriction (IUGR) was defined as birth weight below the sex-specific 5th percentile of weights for gestational age, decreased amniotic fluid volume, or abnormal Doppler. Macrosomia was defined as birth weight above the sex-specific 95th percentile of weights for gestational age.

The Statistical Package for the Social Sciences (SPSS) (Version 12.0.1. for Windows, SPSS Inc.) was used for all data analyses. Demographic characteristics were expressed as means (±standard deviation) and range. The outcome variables were expressed as the absolute number (percentage). *P* value was based on Fisher's exact test for categorical data and the Mann-Whitney *U* test for quantitative variables. Univariate logistic regression analysis was performed to compute odds ratios with 95% confidence intervals of women in Groups 1, 2, and 3 versus nonanemic women (Group 4) for well-known adverse maternal and perinatal outcomes. No correction for multiple testing was performed when comparing single groups with nonanemic women. Those *P* values are only descriptive.

## 3. Results

The demographic and clinical characteristics are shown in Table 1. Iron deficiency anemia was observed in 6.5%, depleted iron stores in 32.2%, and anemia for other reasons in 11.8%. The mean gestational age at study enrolment was 16.3 ± 1.4 weeks. The mean hemoglobin concentration at enrolment was 11.8 ± 0.9 g/dL and serum ferritin was 33.5 ± 27.8 *μ*g/L. Out of 70 anemic women, only mild anemia was observed at enrolment. A higher parity was observed in women with iron deficiency anemia and with depleted iron stores (Table 1). Women in Groups 1, 2, and 3 came more often from former Yugoslavia and developing countries than women in Group 4 (*P* = 0.001).

The maternal outcomes are shown in Table 2. The mean hemoglobin level before delivery was 12.1 ± 1.2 g/dL (7.9–15.4). The prevalence of anemia before delivery was 9.7%, namely, mild anemia in 8.8% and moderate anemia in 0.9% (Hb < 9.0 g/dL). Although iron therapy was given in anemic women, a significantly lower Hb before delivery was observed in these women (*P* = 0.001). There was also a significant difference of hemoglobin concentration before delivery between women with depleted iron stores and normal women (*P* = 0.005). There was no increase of maternal complications in women with anemia and in women with depleted iron stores.

The mean gestational age at delivery was 38.7 ± 2.9 weeks (25–42) and birth weight was 3320 ± 646 g (730–5250) (Table 3). Preterm delivery was observed in 7.6% (29/382), low birth weight in 8.1% (31/382), and perinatal mortality in 0.5% (2/382). There was a significant difference of meconium stained amniotic fluid between women with depleted iron stores and nonanemic women (1/123 versus 14/189) (*P* = 0.006) (Table 3). No difference of low birth weight, IUGR, preterm delivery, or PPROM between anaemic and nonanemic women was ascertained. Macrosomia was more often in women with iron deficiency anemia and with depleted iron stores (16% and 11.4%).

The logistic regression analysis showed that anemia and depleted iron stores are not significant risk factors for adverse pregnancy outcome (Table 4). The upper limits of the 95% confidence intervals of the odds ratios for preterm delivery, LBW, IUGR, and caesarean section showed that mild anemia and depleted iron stores are not associated with those adverse outcomes in iron supplemented women. Placental abruption, abnormal placentation, and puerperal infection were too rare to draw any conclusions.

## 4. Discussion

The prevalence of anemia and depleted iron stores in the present study was 50.5% (193/382), namely, anemia in 18.3% (70/382) and depleted iron stores in 32.2% (123/382). Our results are in accordance with other studies performed in European countries [15]. A higher parity was observed in women with iron deficiency anemia and depleted iron stores. Depleted iron stores were higher in women from former Yugoslavia and anemia for other reasons in women from developing countries.

Mild anemia and depleted iron stores detected early in pregnancy were not associated with adverse outcomes in iron supplemented women. In our study, macrosomia was more often in nondiabetic women with iron deficiency anemia (16.0%). Early nonexcessive placental hyperplasia in women with mild anemia might lead to increased nutrition support in later pregnancy if a stress situation is experienced. To our knowledge, no studies exist observing the increased prevalence of macrosomia in women with iron deficiency anemia.

There is a lot of controversial information about anemia in pregnancy and adverse outcomes. Two points are important for assessment of this relationship: the gestational age at which the determination of hemoglobin is performed and the degree of anemia.

Hemoglobin and hematocrit decline due to physiologic expansion of the plasma volume throughout the 1st and 2nd trimesters [7]. Plasma volume expansion reaches its lowest point late in the second to early in the third trimester and then rises again nearer to term. It is thus becoming clear that the best time to detect any risk associated with maternal anemia may be early in pregnancy. This was also confirmed in the following current studies [10, 16]. Any estimation of hemoglobin concentration taken after 20 weeks' gestation will be reasonably representative of the fall induced by pregnancy [17]. The mean gestational age at enrolment in our study was 16 weeks.

The second important point is the degree of anemia in pregnancy. This is the reason why there is a lot of controversial information about the relationship between anemia and adverse outcomes. The extensive literature review presented strong evidence for an association between maternal hemoglobin and birth weight as well as between maternal hemoglobin and preterm delivery [18]. Mild anemia, which was present in our study, was not associated with adverse outcomes in iron supplemented women. Therefore, we assume that iron supplementation had a protective effect on adverse outcome. On the other hand, severe maternal anemia, particularly in the first trimester, is associated with adverse outcomes, namely, preterm birth, low birth weight, intrauterine growth restriction, low Apgar score, and operative deliveries [5, 9, 10, 16, 19, 20]. The association between the degree of anemia and adverse outcome was investigated by many studies, in which this association was confirmed [5, 9, 10, 21]. Extremely high maternal mortality (6.2%) and perinatal mortality (60%) were determined in the study by Patra et al., in which severe maternal anemia was determined in the third trimester [20].

A meta-analysis of studies on the association between hemoglobin concentration and adverse outcome, conducted between 1985 and 1998, showed that maternal anemia during early pregnancy is associated with slightly increased preterm delivery but not with significantly increased low birth weight or with fetal growth restriction [16]. This meta-analysis did not consider the degree of anemia nor did it use different parameters for the definition of anemia. Generally, the hematological parameters and criteria for anemia differ widely. Some authors use hematocrit <33% as criteria for anemia [22], and others use hemoglobin concentration with a different cut-off, namely, less than 11.0 g/dL, 10.5, or 10.0 g/dL [5, 6, 8–10, 17, 19]. We defined anemia as hemoglobin concentration of 11.0 g/dL or less, since we previously saw high intraindividual variations between Hb 10.5 and 11.0 g/dL and there was a large group of women with hemoglobin between 10.5 and 10.9 g/dL (39/382; 10.2%).

The limitation of our study is the absence of CRP determination, since ferritin is a marker of inflammation. Consequently, high serum ferritin could actually be a false positive in patients with inflammation. The second disadvantage is the lack of any comparison of our results with an untreated group of anemic women. However, if we compare our results with other studies, we can say that iron supplementation had a protective effect on adverse pregnancy outcome. Since we wished to simulate normal supplementation, the women did not have to return any residual supplementation. Thus, compliance was not monitored in this study.

Systematic iron prophylaxis and iron-folic acid supplementation during pregnancy has been debated [23–26]. The first choice in the prophylaxis of iron deficiency anemia for almost all women is oral iron replacement because of its effectiveness, safety, and low cost [24]. However, in practice, physicians are frequently faced with poor compliance, which can lead to anemia. The second choice is intravenous iron supplementation with no drug-related serious adverse effects [25]. The commonly cited disadvantages of intravenous iron supplementation are high cost and the invasive nature of the procedure.

We recommend screening for hemoglobin and iron status in early pregnancy. When there is a good compliance with iron supplementation and the pregnancy is uncomplicated, there is no need for hematological tests during further prenatal visits, even in cases of mild iron deficiency anemia and depleted iron stores detected in early pregnancy. One clear set of hematological test results early in pregnancy indicates that there is no increased risk of adverse maternal and perinatal outcomes due to mild iron deficiency anemia and depleted iron stores in iron supplemented women; further testing later in pregnancy is therefore superfluous.

## 5. Conclusions

Mild anemia and depleted iron stores detected early in pregnancy were not associated with adverse maternal and perinatal outcomes.

## Figures and Tables

**(a):** 

	Group 1	Group 2	Group 3	Group 4	All women
Pregnant women	25/382 (6.5)	123/382 (32.2)	45/382 (11.8)	189/382 (49.5)	382
Maternal age (years)	30.3 ± 5.9 (21.2–38.7)	29.7 ± 5.7 (21.2–44.9)	30.1 ± 6.3 (20.2–41.9)	30.8 ± 5.9 (18.9–44.3)	30.3 ± 5.9 (18.9–44.9)
Gravidity	2.8 ± 1.8 (1–7)	2.4 ± 1.4 (1–7)	2.3 ± 1.5 (1–8)	2.3 ± 1.7 (1–14)	2.4 ± 1.6 (1–14)
=1	7/25 (28.0)	36/123 (29.2)	18/45 (40.0)	74/189 (39.2)	135/382 (35.3)
2–4	14/25 (56.0)	77/123 (62.6)	24/45 (53.3)	100/189 (52.9)	215/382 (56.3)
≥5	4/25 (16.0)	10/123 (8.2)	3/45 (6.7)	15/189 (7.9)	32/382 (8.4)
Parity	2.3 ± 1.3 (1–5)	1.9 ± 0.9 (1–5)	1.7 ± 0.8 (1–4)	1.7 ± 1.0 (1–6)	1.8 ± 0.9 (1–6)
=1	9/25 (36.0)	43/123 (35.0)	22/45 (48.9)	101/189 (53.4)	175/382 (45.8)
2-3	12/25 (48.0)	72/123 (58.5)	22/45 (48.9)	78/189 (41.3)	184/382 (48.2)
≥4	4/25 (16.0)	8/123 (6.5)	1/45 (2.2)	10/189 (5.3)	23/382 (6.1)
Gestational age at delivery (wk)	38.6 ± 2.3 (30–41)	38.9 ± 1.9 (29–42)	38.7 ± 1.8 (33–41)	38.7 ± 2.6 (25–42)	38.7 ± 2.9 (25–42)
<37	2/25 (8)	7/123 (5.7)	2/45 (4.4)	18/189 (9.5)	29/382 (7.6)
37–42	23/25 (92)	116/123 (94.3)	43/45 (95.6)	171/189 (90.5)	353/382 (92.4)
Gestational age at enrolment (wk)	16.3 ± 1.3 (15–19)	16.4 ± 1.3 (15–20)	16.4 ± 1.3 (15–19)	16.2 ± 1.2 (15–19)	16.3 ± 1.4 (15–20)
BMI (kg/m^2^)	22.9 ± 4.8 (18.2–35.5)	23.5 ± 5.5 (15.4–50.9)	24.1 ± 4.3 (17.6–32.6)	24.2 ± 5.1 (17.8–45.2)	23.9 ± 5.1 (15.4–50.9)
Origin of mother					
Europe + North America	2/25 (8.0)	37/123 (30.1)	7/45 (15.6)	76/189 (40.2)	122/382 (31.9)
Former Yugoslavia	12/28 (48.0)	49/123 (39.8)	16/45 (35.6)	50/189 (26.5)	127/382 (33.2)
Developing countries	11/25 (44.0)	37/123 (30.1)	22/45 (48.9)	63/189 (33.3)	133/382 (34.8)

Data expressed as mean ± s.d. (range) or number (%).

**(b):** 

*P* value	1 versus 4	2 versus 4	3 versus 4	1, 2, and 3 versus 4
Maternal age	0.827	0.068	0.336	0.082
Gravidity	0.121	0.144	0.913	0.153
Parity	0.029^*^	0.005^**^	0.797	0.006^*^
Gestational age at delivery	0.633	0.687	0.485	0.919
BMI	0.19	0.383	0.941	0.31
Origin of mother	0.05	0.038^*^	0.008^*^	0.001^**^

^*^ *P* value < 0.05; ^**^ *P* value < 0.005.

Group 1: iron deficiency anemia.

Group 2: depleted iron stores.

Group 3: anemia for other reasons.

Group 4: normal status.

**(a):** 

	Group 1	Group 2	Group 3	Group 4	All women
Hb at delivery (g/dL)	10.9 ± 1.1 (8.8–12.7)	12.1 ± 1.2 (7.9–15.4)	11.3 ± 0.9 (9.1–12.9)	12.5 ± 0.9 (9.5–15.1)	12.1 ± 1.2 (7.9–15.4)
Placental abruption	0/25 (0.0)	1/123 (0.8)	0/45 (0.0)	1/189 (0.5)	2/382 (0.5)
Abnormal placentation	2/25 (8.0)	4/123 (3.3)	0/45 (0.0)	2/189 (1.1)	8/382 (2.1)
Preeclampsia, eclampsia	0/25 (0.0)	1/123 (0.8)	3/45 (6.7)	4/189 (2.1)	8/382 (2.1)
Cardiac failure	0/25 (0.0)	0/123 (0.0)	0/45 (0.0)	0/189 (0.0)	0/382 (0.0)
Delivery modus					
Nonoperative vaginal delivery	16/25 (64.0)	69/123 (56.1)	23/45 (51.1)	91/189 (48.1)	199/382 (52.1)
Operative vaginal delivery	1/25 (4.0)	9/123 (7.3)	3/45 (6.7)	31/189 (16.4)	44/382 (11.5)
Caesarean section prim.	6/25 (24.0)	25/123 (20.3)	14/45 (31.3)	37/189 (19.6)	82/382 (21.5)
Caesarean section sec.	2/25 (8.0)	20/123 (16.3)	5/45 (11.1)	30/189 (15.9)	57/382 (14.9)
Postpartum hemorrhage	1/25 (4.0)	7/123 (5.7)	6/45 (13.3)	21/189 (11.1)	35/382 (9.2)
Puerperal sepsis	0/25 (0.0)	0/123 (0.0)	0/45 (0.0)	0/189 (0.0)	0/382 (0.0)
Puerperal infection	0/25 (0.0)	6/123 (4.9)	1/45 (2.2)	6/189 (3.2)	13/382 (3.4)
Urinary tract infection	0/25 (0.0)	1/123 (0.8)	0/45 (0.0)	1/189 (0.5)	2/382 (0.5)
Wound infection	0/25 (0.0)	1/123 (0.8)	0/45 (0.0)	2/189 (1.1)	3/382 (0.8)
Mastitis	0/25 (0.0)	2/123 (1.6)	1/45 (2.2)	2/189 (1.1)	5/382 (1.3)
Endometritis	0/25 (0.0)	1/123 (0.8)	0/45 (0.0)	0/189 (0.0)	1/382 (0.3)
Thrombophlebitis	0/25 (0.0)	1/123 (0.8)	0/45 (0.0)	1/189 (0.5)	1/382 (0.3)
Infection of unclear etiology	0/25 (0.0)	1/123 (0.8)	0/45 (0.0)	0/189 (0.0)	1/382 (0.3)
Subinvolution	1/25 (4.0)	3/123 (2.4)	0/45 (0.0)	2/189 (1.1)	6/382 (1.6)

**(b):** 

*P* value	1 versus 4	2 versus 4	3 versus 4	1, 2, and 3 versus 4
Hb at delivery	0.001^***^	0.005^**^	0.001^***^	0.001^***^
Placental abruption	1	1	1	1
Abnormal placentation	0.068	0.217	1	0.284
Preeclampsia, eclampsia	1	0.652	0.132	1
Delivery modus	0.211	0.125	0.157	0.021^*^
Postpartum hemorrhage	0.482	0.11	0.613	0.217
Puerperal infection	1	0.55	1	1
Subinvolution	0.312	0.386	1	0.685

^*^ *P* value < 0.05; ^**^ *P* value < 0.005; ^***^ *P* value < 0.001.

**(a):** 

	Group 1	Group 2	Group 3	Group 4	All women
Gestational age at delivery (wk)	38.6 ± 2.3	38.9 ± 1.9	38.7 ± 1.8	38.7 ± 2.6	38.7 ± 2.9 (25–42)
Birth weight (g)	3412 ± 605	3369 ± 625	3218 ± 546	3299 ± 687	3320 ± 646 (730–5250)
Meconium in amniotic fluid	3/25 (12.0)	1/123 (0.8)	3/45 (6.7)	14/189 (7.4)	21/382 (5.5)
LBW	2/25 (8.0)	7/123 (5.7)	3/45 (6.7)	19/189 (10.1)	31/382 (8.1)
IUGR	0/25 (0.0)	7/123 (5.7)	3/45 (6.7)	12/189 (6.3)	22/382 (5.8)
Macrosomia	4/25 (16.0)	14/123 (11.4)	0/45 (0.0)	15/189 (7.9)	33/382 (8.6)
Preterm delivery	2/25 (8.0)	7/123 (5.7)	2/45 (4.4)	18/189 (9.5)	29/382 (7.6)
PPROM	1/25 (4.0)	2/123 (1.6)	0/45 (0.0)	7/189 (3.7)	10/382 (2.6)
Still birth	0/25 (0.0)	0/123 (0.0)	1/45 (2.2)	0/189 (0.0)	1/382 (0.3)
Neonatal death	0/25 (0.0)	0/123 (0.0)	0/45 (0.0)	1/189 (0.5)	1/382 (0.3)
NICU admissions	0/25 (0.0)	0/123 (0.0)	0/45 (0.0)	1/189 (0.5)	1/382 (0.3)
Apgar score at 1′	8.1 ± 0.8 (6–9)	7.9 ± 0.9 (3–9)	7.9 ± 1.5 (0–10)	7.7 ± 1.3 (1–9)	7.9 ± 1.2 (0–10)
Apgar score at 5′	8.8 ± 0.7 (7–10)	9.0 ± 0.5 (7–10)	8.8 ± 1.5 (0–10)	8.9 ± 0.9 (3–10)	8.9 ± 0.9 (0–10)
Apgar score <5 at 5′	0/25 (0.0)	0/123 (0.0)	1/45 (2.2)	3/189 (1.6)	4/382 (1.0)

**(b):** 

*P* value	1 versus 4	2 versus 4	3 versus 4	1, 2, and 3 versus 4
Meconium in amniotic fluid	0.428	0.006^*^	1	0.12
LBW	1	0.211	0.583	0.192
IUGR	0.368	1	1	0.666
Macrosomia	0.25	0.324	0.82	0.717
Preterm delivery	1	0.287	0.38	0.179
PPROM	1	0.491	0.351	0.216
Still birth	NoS	NoS	0.192	1
Neonatal death	1	1	1	0.495
NICU admissions	1	1	1	0.495
Apgar score <5 at 5′	1	0.281	0.577	0.368

^*^ *P* value < 0.05.

NoS: no statistics are computed because variable is a constant.

**Table 4:** Logistic regression analysis of adverse outcomes among anemic women and women with depleted iron stores (Groups 1, 2, and 3) versus nonanemic women (Group 4).

Adverse outcome	Groups 1, 2, and 3	Group 4	O.R. (95% CI)	*P* value
	(*n* = 193)	(*n* = 189)		
Preterm delivery	11/193 (5.7)	18/189 (9.5)	0.58 (0.26–1.25)	0.162
LBW	12/193 (6.2)	19/189 (10.1)	0.59 (0.28–1.26)	0.174
IUGR	10/193 (5.2)	12/189 (6.4)	0.81 (0.34–1.91)	0.625
Caesarean section	72/193 (37.3)	67/189 (35.5)	0.92 (0.60–1.43)	0.75
